# Long-term results of sirolimus treatment in lymphangioleiomyomatosis: a single referral centre experience

**DOI:** 10.1038/s41598-021-89562-0

**Published:** 2021-05-13

**Authors:** Eva Revilla-López, Cristina Berastegui, Alejandra Méndez, Berta Sáez-Giménez, Victoria Ruiz de Miguel, Manuel López-Meseguer, Victor Monforte, Carlos Bravo, Miguel Angel Pujana, Maria Antonia Ramon, Susana Gómez-Ollés, Antonio Roman, The Vall d’Hebron Multidisciplinary Cystic Lung Disease Group, Irene Bello, Rosa Burgos, Roser Escobar, Carla Ferrándiz-Pulido, Alba Gómez, Esther Pallisa, Gloria Palomares, Sabina Salicrú, Ana Lucía Sánchez Martínez, Irene Sansano, Joana Sellarés

**Affiliations:** 1 Lung Transplant Program, Department of Pulmonology, Departament de Medicina, Vall d’Hebron Institut de Recerca (VHIR), Hospital Universitari Vall d’Hebron, Universitat Autònoma de Barcelona (UAB), Passeig Vall d’Hebron, 119-129, 08035 Barcelona, Spain; 2 CIBER de Enfermedades Respiratorias (CIBERES), Instituto de Salud Carlos III, Madrid, Spain; 3 Program Against Cancer Therapeutic Resistance (ProCURE), Breast Cancer and Systems Biology, Catalan Institute of Oncology (ICO), Bellvitge Institute for Biomedical Research (IDIBELL), L’Hospitalet del Llobregat, Catalonia, Spain; 4 Lung Transplant Unit, Department of Thoracic Surgery, Hospital Universitari Vall d’Hebron, Universitat Autònoma de Barcelona (UAB), Barcelona, Spain; 5 Nutritional Support Unit, Hospital Universitari Vall d’Hebron, Universitat Autònoma de Barcelona (UAB), Barcelona, Spain; 6 Department of Dermatology, Hospital Universitari Vall d’Hebron, Universitat Autònoma de Barcelona (UAB), Barcelona, Spain; 7 Physical Medicine and Rehabilitation Department, Hospital Universitari Vall d’Hebron, Universitat Autònoma de Barcelona (UAB), Barcelona, Spain; 8 Department of Radiology, Hospital Universitari Vall d’Hebron, Universitat Autònoma de Barcelona (UAB), Barcelona, Spain; 9 Department of Psychiatry, Hospital Universitari Vall d’Hebron, Universitat Autònoma de Barcelona (UAB), Barcelona, Spain; 10 Department of Obstetrics and Gynaecology, Hospital Universitari Vall d’Hebron, Universitat Autònoma de Barcelona (UAB), Barcelona, Spain; 11 Department of Pathology, Hospital Universitari Vall d’Hebron, Universitat Autònoma de Barcelona (UAB), Barcelona, Spain; 12 Nephrology Department, Hospital Universitari Vall d’Hebron, Universitat Autònoma de Barcelona (UAB), Barcelona, Spain

**Keywords:** Respiratory tract diseases, Diagnosis, Drug therapy

## Abstract

There are few published data on long-term treatment with sirolimus in lymphangioleiomyomatosis (LAM). The objective of this study was to describe the long-term effect of sirolimus in a series of LAM patients followed up in a referral centre, focusing on pulmonary function. We retrospectively reviewed a series of 48 patients with LAM diagnosed, followed up and treated with sirolimus in a single centre. Response to sirolimus was evaluated at 1 and 5 years. A negative sirolimus response was defined as an FEV_1_ decline greater than − 75 ml/year. A mixed-effects model was used to estimate the longitudinal changes in FEV_1_ (average slope), both as absolute (ml/year) and as predicted values (%predicted/year). From a total of 48 patients, 9 patients underwent lung transplantation and 4 died during the study. Mean (95% CI) FEV_1_ slope over 5 years was − 0.14 (− 26.13 to 25.85) ml/year in the whole LAM group, 42.55 (14.87 to 70.22) ml/year in the responder group, − 54.00 (− 71.60 to − 36.39) ml/year in the partial responder group and − 84.19 (− 113.5 to − 54.0) ml/year in the non-responder group. After 5 years of sirolimus treatment 59% had a positive response, 30% had a partial response and 11% had a negative response. Our study found that sirolimus treatment had a positive long-term effect on most LAM patients.

## Introduction

Lymphangioleiomyomatosis (LAM) is a rare, slowly progressive disease characterized by the proliferation of abnormal smooth muscle-like cells named LAM cells^1^. This cell proliferation is associated with cystic lung disease, chylous fluid accumulations and abdominal angiomyolipomas and lymphangioleiomyomas^2^. LAM affects almost exclusively adult women and occurs sporadically (S-LAM) or in association with the tuberous sclerosis complex (TSC-LAM). TSC-LAM is caused by mutations in two tumour suppressor genes: *TSC1* and *TSC2*, with consequent loss of function of hamartin and tuberin proteins, respectively^3,4^. S-LAM, by contrast, is not hereditary. Recently, a genome-wide association study was performed and the data suggested that NR2F2 expression may have a role in S-LAM^5^.

In recent years, mTOR inhibitors have emerged as a therapeutic option for LAM. Before 2011, multiple non-evidence-based medical treatments were described, such as hormonal manipulation, oophorectomy or tamoxifen^6,7^, and most eligible LAM patients who developed respiratory failure were referred for lung transplant (LT). After 2011, with the publication of the Multicentre International LAM Efficacy of Sirolimus (MILES) trial^8^ and its approval by the *Food and Drug Administration* in 2015, sirolimus was introduced as the first effective treatment in these patients. However, long-term results of sirolimus treatment are scarce^9^.

Our institution developed the first lung transplant program in Spain in 1990. The programme receives national patient referrals with LAM: this provided an impetus to adopt the most recent advances in LAM treatment. Sirolimus was introduced, as an off-label use, in LAM patients in November 2007 following the publication of the first trial in renal angiomyolipomas^10^. The objective of the present study was to review the long-term effect of sirolimus in a series of LAM patients followed up in a referral centre, focusing on pulmonary function.

## Patients and clinical assesment

The study retrospectively included all patients with LAM diagnosed, treated with sirolimus and followed up in a tertiary referral centre from January 1990 to January 2020. Data compiled included clinical findings, diagnostic methods, treatment, lung function, presence of tuberous sclerosis and extrapulmonary involvement such as renal angiomyolipoma, pleural or abdominal complications. The Ethics Committee of the Vall d’Hebron University Hospital (Barcelona, Spain BSG-SIR-2019–01) approved the study. Informed consent was waived due to retrospective nature of the study by The Ethics Committee of the Vall d’Hebron University Hospital. All methods in this study were performed in accordance with the relevant guidelines and regulations.

Pulmonary function tests were performed according to European Respiratory Society guidelines^11^. All FEV_1_ values in our study were obtain without bronchodilators. Patients with LAM underwent spirometric variables and diffusing capacity -at baseline, 1 year, 2 years, 3 years, 4 years and 5 years. Thoracic and abdominal CT scan were performed in all patients. Lung biopsy was performed only in patients with exclusive pulmonary involvement; biopsy was generally avoided if patients had lung cysts associated with renal angiomyolipomas.

Time to event was calculated as the number of years between sirolimus initiation to the date of lung transplantation or date of death, whichever came first.

Patients were classified into two cohorts according to the availability of sirolimus at diagnosis: a historical sirolimus cohort (HSC) of patients for whom sirolimus was not available at diagnosis but who received the drug after 2007 and a contemporary cohort (CC), in whom sirolimus was started immediately after the diagnosis.

### Sirolimus treatment

From November 2007, sirolimus was indicated in the case of FEV_1_ < 80%, angiomyolipoma bigger than 4 cm of diameter or multiple angiomyolipomas. Sirolimus was prescribed at a dose between 1 and 4 mg once daily adjusted to obtain target trough blood levels between 5 and 15 ng/ml measured with liquid chromatography-mass spectrometry. Adverse effects and drug interactions were also recorded.

We defined the sirolimus response based on the rate of FEV_1_ decline in LAM patients without specific treatment, which has been reported as − 75 ml/year^12^. Age-related physiological decline in healthy adults under 60 years old has been reported to be around − 20 ml/year^13–15^. Consequently, improvement, stabilization or loss of FEV_1_ similar to the physiological decline was considered a positive response to sirolimus. Patients who had an FEV_1_ decline rate between − 20 and − 75 ml/year were considered partial responders. Non-responders had an FEV_1_ decline greater than − 75 ml/year. Sirolimus response was assessed at 1 year and long-term response at 5 years. Patients who underwent LT before completing 1 or 5 years of sirolimus treatment were not included in the respective analysis.

Angiomyolipomas’ shrinkage rate was calculated as the change in maximum diameter after 1 year of sirolimus treatment. Patients with a decrease or stabilization in angiomyolipoma size were considered responders.

### Statistical analysis

Mixed-effect models were used to estimate the longitudinal changes in FEV_1_ (average slope), both as absolute (ml/year) and as predicted values (%predicted/year). In these analyses, time was entered as a random effect. We then categorised individuals into three groups: (1) responders (if the individualised estimate was less than − 20 ml/year), (2) partial responders (if the individualised estimate was between − 20 and − 75 ml/year); and (3) non-responders (if the individualised estimate was greater than − 75 ml/year). Qualitative data are expressed as frequencies and percentages. Normally-distributed quantitative data are expressed as mean ± SD; non-normally-distributed data are expressed as median and interquartile range (IQR). The demographic and clinical characteristics of the HSC and CC were compared using ANOVA or chi-square test, as appropriate. Pulmonary function tests (PFT) of non-responders, partial responders and responders to sirolimus were compared using the Mann Whitney test. Time from sirolimus initiation to death/transplantation was assessed using Kaplan–Meier curves. Cox regression analysis was used to compared HSC and CC. The 95% CI was obtained from the mean estimates. A *p*-value < 0.05 was considered statistically significant. Data were analysed using STATA version 14 (StataCorp, College Station, TX, USA) and SPSS version 27 (IBM Corp., Armonk, NY, USA).

## Results

Forty-eight LAM patients diagnosed and followed-up in our outpatient clinic and treated with sirolimus were recruited: 19 (40%) patients correspond to HSC and 29 (60%) to CC. Four (8.3%) of them had LAM associated with TSC. The median age at onset of symptoms and at diagnosis was 36 (30–44) and 38 (34–46) years, respectively. Thirty-nine (81%) patients were pre-menopausal at diagnosis and 9 (19%) were post-menopausal. Three (6%) patients were smokers at diagnosis and 14 (29%) were ex-smokers. Dyspnoea was the most common presenting symptom in 17 (35%) patients followed by pneumothorax in 15 (31%) patients. Mean FEV_1_ at diagnosis was 68 ± 26% of the predicted. On thoracic CT, all patients had multiple cystic images with bilateral distribution, predominantly in the middle and lower lobes. The method of diagnosis was surgical lung biopsy in 28 (58%) patients, clinicoradiological findings in 12 (25%) and transbronchial cryobiopsy in 8 (17%). Three (6%) diagnoses were incidental in asymptomatic patients.

The mean duration of follow-up was 11.7 ± 6.9 years from diagnosis and mean time of sirolimus treatment was 5.6 ± 3.5 years. During follow-up, the most common pleural complications were pneumothorax, which occurred in 8 (17%) patients, chylous effusion in 4 (8%), and transudative pleural effusion in 3 (6%). Nineteen (40%) patients needed chemical pleurodesis and 2 (4%) needed thoracic duct ligation. Twenty (42%) patients had renal angiomyolipoma; 2 of them required embolization and 2 underwent surgery. Other extrapulmonary complications included 5 (10%) cases of abdominal lymphangioleiomyomas.

From the study cohort of 48 patients, 9 (19%) patients underwent lung transplantation (LT). These patients were treated with sirolimus before the LT for a median time of 18 (7–42) months. Pre-sirolimus spirometry demonstrated severe obstruction: mean FVC was 47 ± 15% pred., FEV_1_ was 26 ± 9% pred. and FEV_1_/FVC ratio was 45 ± 11%. Pre-LT spirometry showed a mean FVC of 44 ± 18%, FEV_1_ of 30 ± 15% and FEV_1_/FVC ratio of 43 ± 10%. After LT, patients received tacrolimus, mycophenolate mofetil and steroids as immunosuppression according to the lung transplant unit protocol.

During the study period, 4 (8%) patients died, only one of them had received LT and died from an acute cellular rejection. The other causes of death were respiratory failure, renal failure and cholangiocarcinoma. A comparison between the HSC and CC is presented in Table 1.

**Table 1:** Comparison between historical sirolimus cohort (sirolimus not available at diagnosis) and contemporary cohort.

	Historical sirolimus cohort n = 19	Contemporary cohort n = 29	*p*-value
**Age at diagnosis (years), median (IQR)**	35 (29–38)	43 (36–46)	0.014
**Initial symptom, n (%)**			0.773
Dyspnoea	7 (37)	10 (34)	
Pneumothorax	7 (37)	8 (28)	
Haemoptysis	2 (11)	2 (7)	
Chylothorax	1 (5)	4 (14)	
Asymptomatic	1 (5)	2 (7)	
Cough	0 (0)	1 (3)	
Abdominal pain	0 (0)	2 (7)	
Pleuritic chest pain	1 (5)	0 (0)	
**Diagnostic method, n (%)**			0.042
Surgical lung biopsy	13 (68)	15 (52)	
Clinicoradiological	6 (32)	6 (21)	
Transbronchial cryobiopsy	0 (0)	8 (28)	
**PFT at diagnosis, mean (SD) median (IQR)**			
FVC, ml	2651 (1037) 2890 (1820–3130)	3063 (841) 3070 (2320–3850)	0.137
FVC, % pred	74 (27) 77 (55–91)	80 (20) 81 (69–98)	0.375
FEV_1_, ml	1638 (901) 1610 (740–2300)	2186 (760) 2070 (1670–2770)	0.028
FEV_1_, % pred	57 (28) 59 (30–81)	76 (22) 77 (62–94)	0.009
DL_CO_, %	42 (21) 42 (21–63)	60 (21) 52 (46–79)	0.010
**Suppl. oxygen use, n (%)**	8 (42)	4 (14)	0.027
**Associated TSC, n (%)**	3 (16)	1 (3)	0.130
**AML, n (%)**	12 (63)	8 (28)	0.015
**Pneumothorax during follow-up**			0.025
Number pneumothorax/n patients	21/6	2/2	
**Chylothorax during follow-up**			0.130
Number chylothorax/n patients	4/3	1/1	
**Chemical pleurodesis**	11 (58)	8 (28)	0.044
**Thoracic duct ligation**	0 (0)	2 (7)	0.242
**Follow-up (years), median (IQR)**	17 (14–22)	8 (5–10)	< 0.001
**LT, n (%)**	8 (42)	1 (3)	< 0.001
**Death, n (%)**	3 (16)	1 (3)	0.130

*IQR* interquartile range, *SD* standard deviation, *PFT* pulmonary function test, *TSC* tuberous sclerosis complex, *AML* angiomyolipoma, *LT* lung transplantation.

**Figure 1:**
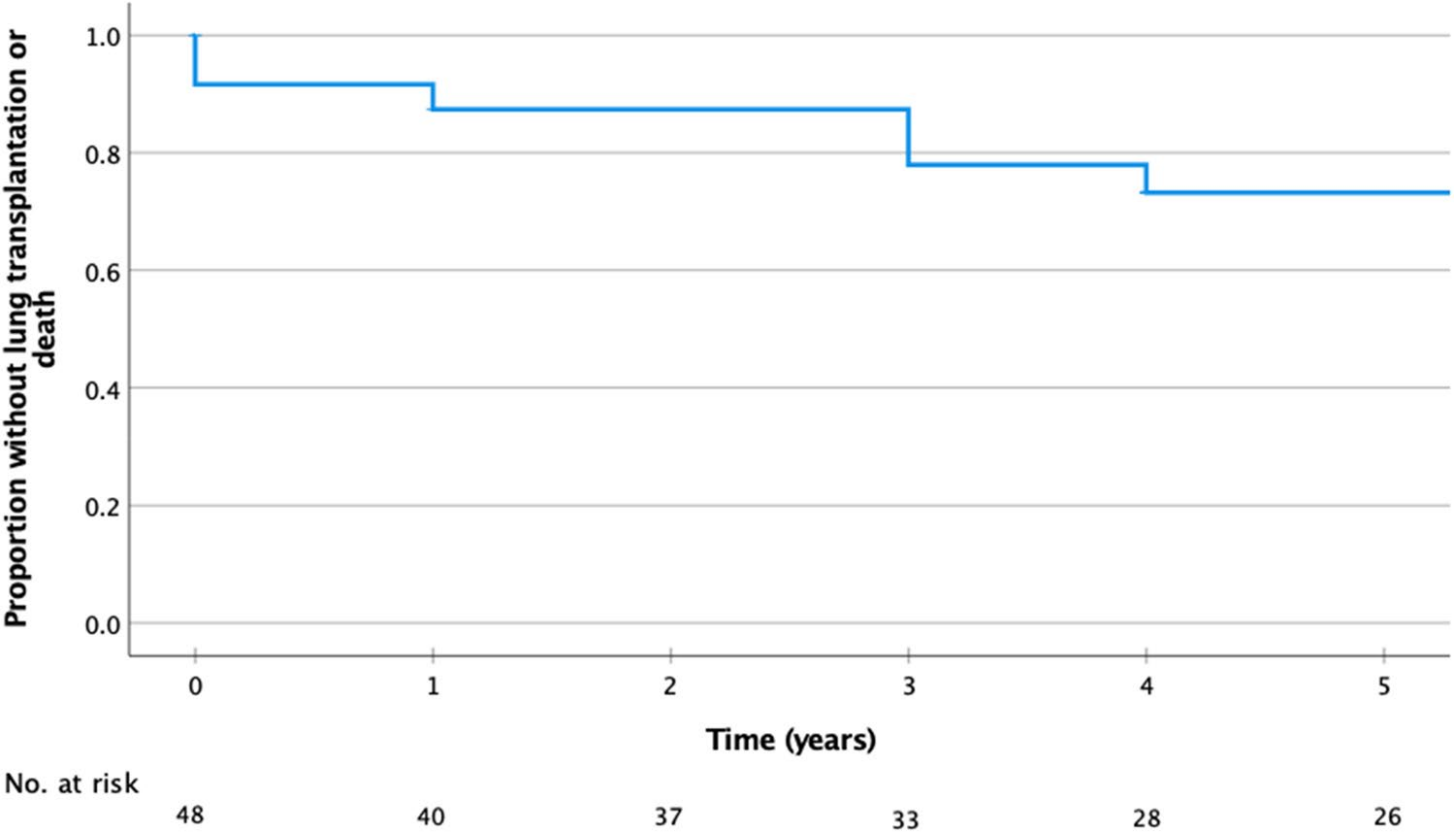
Kaplan–Meier survival curve free from death/transplantation from the time of sirolimus initiation. Mean time of sirolimus treatment was 5.6 ± 3.5 years.

Survival rates free from death and/or transplantation at 1- and 5-years after sirolimus initiation were 87% and 73% respectively (Fig. 1).

**Figure 2:**
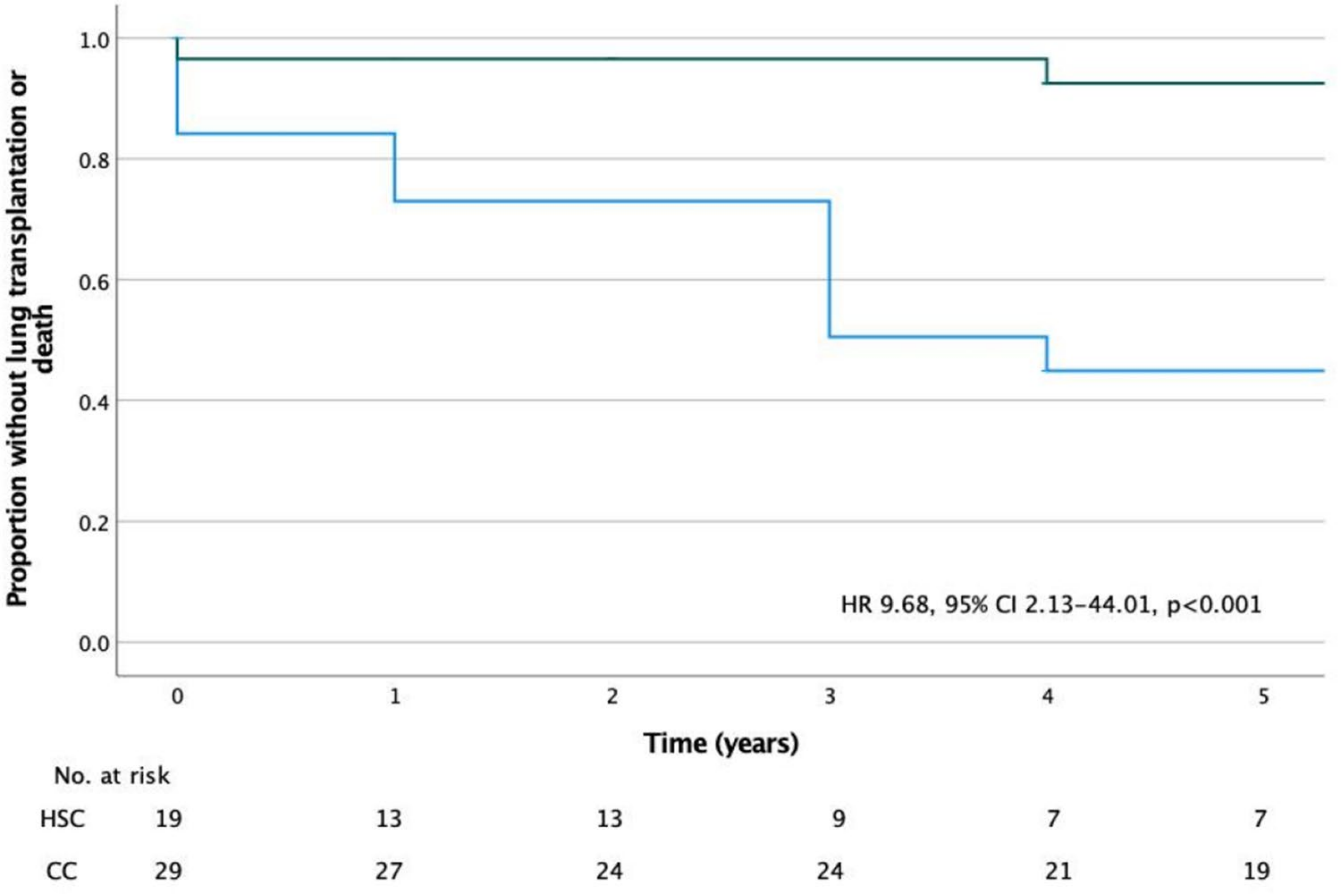
Kaplan Meier survival curve free from death/transplantation from the time of sirolimus initiation. HSC (blue) and CC (green).

HSC patients presented a significantly higher long-term risk of death and/or LT compared to CC (Fig. 2).

### Sirolimus treatment

From November 2007, 48 patients with LAM were treated at our hospital with sirolimus. Twenty-eight (58%) patients initiated sirolimus because of FEV_1_ < 80%, 4 (8%) because of AML and 16 (33%) because of both. Mean sirolimus dose was 2.2 ± 0.8 mg and mean blood trough levels were 7.5 ± 2.7 ng/ml.

Of note, only one patient from the HSC developed pneumothorax under sirolimus treatment, while the others developed this complication before they started treatment. No patients developed chylothorax under sirolimus treatment (1 patient from the CC experienced the complication before initiating treatment).

In the HSC, the median time between onset of symptoms and starting sirolimus was 12 (4–15) years. In the CC, the median time from the onset of symptoms to starting sirolimus was 1 (0–3) years. The mean spirometry values before sirolimus in the HSC and CC were: FVC 62 ± 25% pred. versus 84 ± 21% pred respectively (*p* = 0.002), FEV_1_ 41 ± 21% pred. versus 74 ± 22% pred. (*p* < 0.001), DL_CO_ 40 ± 22% versus 55 ± 17% (*p* = 0.228).

#### Safety

Side effects were recorded in 16 (33%) patients and tended to occur shortly after starting treatment and decrease over time. The most common side effect was oral aphthous ulcers in 8 (17%), ovarian cysts in 3 (6%), severe increase in serum cholesterol in 2 (4%), liver enzyme abnormalities in 2 (4%), anasarca in 1 (2%) and leg swelling in 1 (2%). Only 1 patient discontinued sirolimus treatment due to fluid retention and in 3 cases sirolimus was temporarily withdrawn due to oral aphthous ulcers.

#### Efficacy

**Figure 3:**
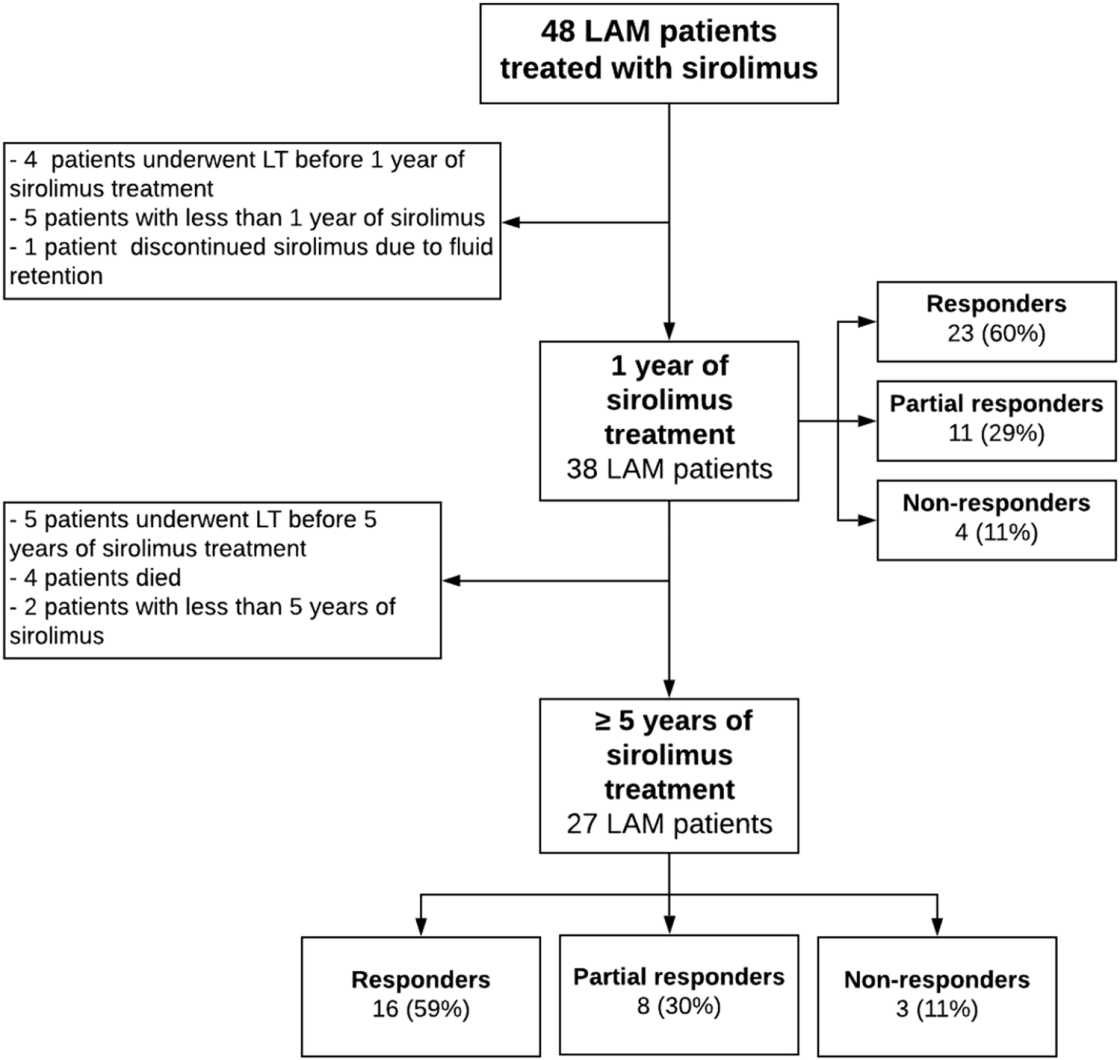
Response to sirolimus after 1 and 5 years of treatment.

At the end of the study, of 48 sirolimus-treated patients, 38 (79%) had had treatment for 1 year and 27 (56%) had had a minimum of 5 years’ treatment (Fig. 3).

After 1 year, a positive response to sirolimus was observed in 23 (60%) patients and a partial response in 11 (29%) (Fig. 3). Long-term efficacy of sirolimus was studied in 27 patients who had received 5 years or more of sirolimus treatment: 59% were responders, 30% were partial responders and 11% non-responders. There were no differences in FVC, FEV_1_ or DL_CO_ at diagnosis or before sirolimus treatment between responders, partial responders and non-responders; see Table 2. However, significantly differences were observed in FEV_1_ after 5-years of sirolimus treatment having non-responders lower values.

**Table 2:** Patient’s characteristics according to sirolimus response at 5 years.

Response	Responders (n = 16)	Partial responders (n = 8)	Non-responders (n = 3)	*p* value
**Age at diagnosis (years), mean (SD)**	35 (6)	50 (8)	39 (5)	< 0.01
**Age at start of sirolimus (years), mean (SD)**	38 (6)	50 (8)	42 (3)	0.001
**Pregnancy before diagnosis, n (%)**	9 (31)	4 (50)	3 (67)	0.300
**Post-menopausal at sirolimus initiation, n (%)**	2 (13)	5 (63)	0 (0)	0.017
**Pneumothorax, n (%)**	10 (63)	3 (38)	2 (67)	0.468
**Chylothorax, n (%)**	4 (25)	0 (0)	0 (0)	0.199
**Pleurodesis, n (%)**	8 (50)	2 (25)	1 (33)	0.483
**FVC (ml), mean (SD)** before sirolimus	2959 (825)	2854 (521)	3187 (479)	0.794
**FEV**_**1**_** (ml), mean (SD)** before sirolimus	1961 (632)	1980 (602)	1730 (789)	0.831
**DL**_**CO**_** (%), mean (SD)** before sirolimus	52 (14)	52 (16)	38 (24)	0.411
**Mean sirolimus blood trough levels**	8.2 (2.9)	6.6 (2.7)	7.1 (1.1)	0.374

*IQR* interquartile range, *SD* standard deviation.

Of note, from the responders group, four patients developed chylothorax and only one patient was treated by pleurodesis before sirolimus was initiated. In the other 3 patients, sirolimus was started due to the chylothorax.

**Figure 4:**
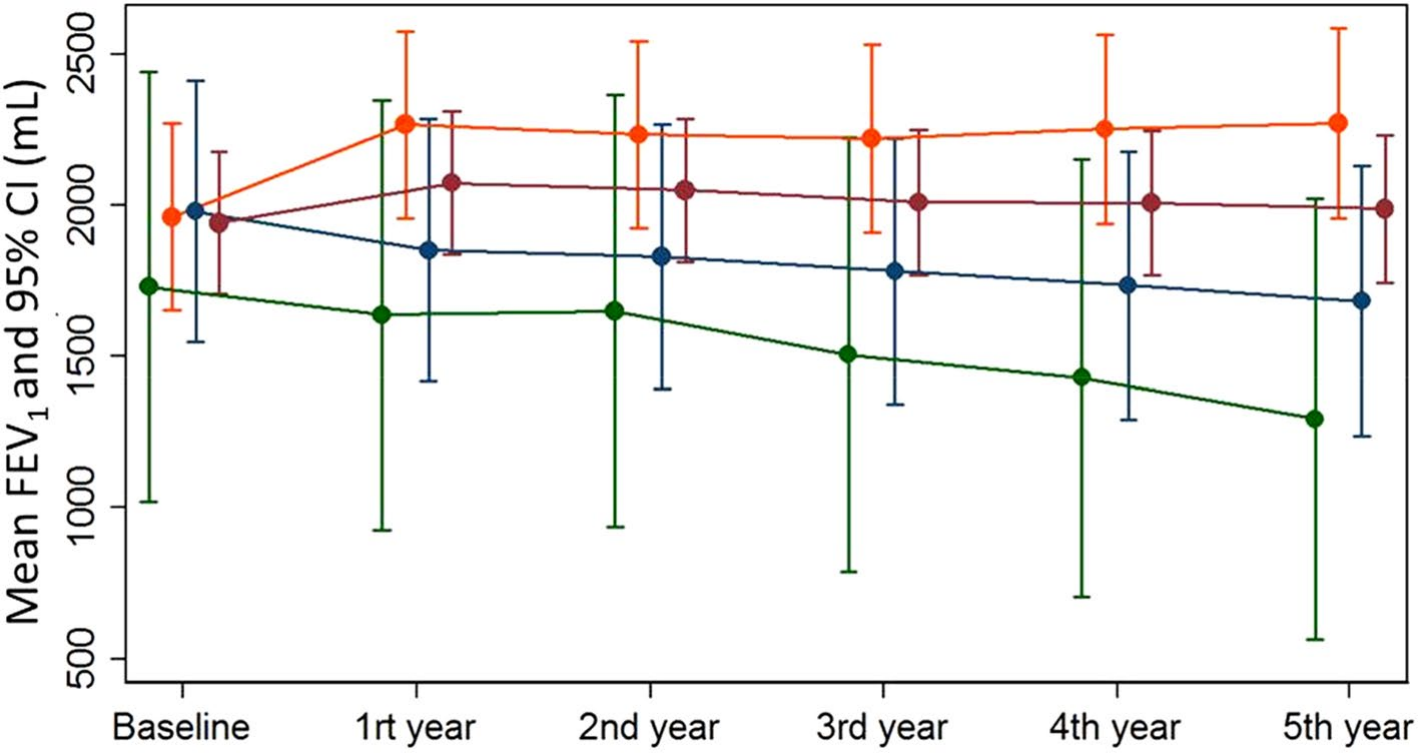
Linear prediction of longitudinal changes in FEV_1 _(ml) and predictive margins with 95% CI using mixed effect models, in all patients (red), responders (yellow), partial responders (blue) and non-responders (green).

Mean (95% CI) FEV_1_ slope over 5 years was − 0.14 (− 26.13 to 25.85) ml/year in the whole LAM group, 42.55 (14.87 to 70.22) ml/year in the responder group, − 54.00 (− 71.60 to − 36.39) ml/year in the partial responder group and − 84.19 (− 113.5 to − 54.0) ml/year in the non-responder group. When the 3 patients from the responder group in whom sirolimus was started due to chylothorax were excluded, the FEV_1_ slope was 28.48 (9.05–47.91) ml/year. When using FEV_1_ as percentage of predicted (%pred) values, mean (95% CI) FEV_1_ slope over 5 years was 0.43 (− 0.55 to 1.41) %pred/year in the whole LAM group, 2.17 (1.25 to 3.09) %pred/year in the responder group, − 1.78 (− 2.50 to − 1.05) %pred/year in the partial responder group and − 2.96 (− 4.05 to − 1.87) %pred/year in the non-responder group. Table 3 summarises the differences in FEV_1_ at 1 year and 5 years and Fig. 4 shows the linear prediction of longitudinal changes in FEV_1_ (ml).

**Table 3:** FEV_1_ response to sirolimus in 27 patients treated with sirolimus for 5 years.

	All (n = 27)	Responders (n = 16)	Partial responders (n = 8)	Non-responders (n = 3)	*p* value
Pre-sirolimus FEV_1_, ml	1941 (1704–2177)	1961 (1653–2268)	1980 (1545–2414)	1730 (1020–2440)	0.831
FEV_1_ at 1 year, ml	2073 (1836–2310)	2266 (1959–2574)	1851 (1416–2286)	1637 (926–2347)	0.185
FEV_1_ at 2-year, ml	2050 (1812–2287)	2234 (1925–2542)	1831 (1395–2268)	1650 (937–2363)	0.272
FEV_1_ at 3-year, ml	2010 (1771–2249)	2219 (1909–2530)	1781 (1342–2220)	1507 (789–2223)	0.180
FEV_1_ at 4-year, ml	2007 (1766–2247)	2251 (1939–2564)	1734 (1292–2176)	1430 (708–2152)	0.092
FEV_1_ at 5-year, ml	1988 (1745–2231)	2271 (1955–2586)	1683 (1236–2129)	1293 (564–2022)	0.041

Data are shown as Mean (95% CI).

Eleven patients with angiomyolipoma were treated with sirolimus for at least one year. Table 4 shows angiomyolipoma sizes before and after 1 year of sirolimus treatment: 10 (91%) patients showed a response to treatment and only 1 (9%) had an increase in tumour size. During follow-up, no patient presented symptoms related to angiomyolipoma.

**Table 4:** Angiomyolipomas’ shrinkage rate at 1 year.

Patients	Number of AML	AML size before sirolimus treatment (cm)	AML size after 1-year of sirolimus (cm)	FEV_1_ difference at 1-year of sirolimus treatment (ml)	Shrinkage rate (%)
LAM-1	1	13.5 × 7.8	4.2 × 1.5	− 280	− 69
LAM-2	3	2 × 1.5 2 × 2 1.6 × 7	2 × 1.5 2 × 2 1.6 × 7	320	0
LAM-3	1	2 × 1	2 × 1	40	0
LAM-4*	3	5 × 2 2.1 × 1 1 × 0.5	6.2 × 3.3 2.1 × 1 1 × 0.5	230	24
LAM-5	1	0.4 × 0.5	0.4 × 0.5	130	0
LAM-6	2	1 × 1	1 × 1	660	0
LAM-7	1	5.5 × 3.5	4.2 × 2.7	40	− 23
LAM-8	1	0.5 × 0.5	0.5 × 0.5	− 30	0
LAM-9	2	2.6 × 1 2 × 1	2.6 × 1 2 × 1	140	0
LAM-10	1	2 × 1.6	2 × 1.6	90	0
LAM-11	1	6.6 × 4.5	3.3 × 3.7	20	− 50

*AML* angiomyolipoma.

*TSC-LAM.

## Discussion

There are few data on long-term pulmonary function results in LAM patients receiving sirolimus. The main finding from this study was that 59% of sirolimus-treated patients were considered responders because they had a sustained spirometric improvement after 5 years of treatment. The MILES trial found that the FEV_1_ slope in the sirolimus group gained 1 ± 2 ml per month during the first year of treatment^8^. Although this difference was statistically significant between sirolimus and placebo groups, a substantial number of patients receiving sirolimus treatment had a decrease in FEV_1_ during this period. One recent Japanese series of 63 patients treated with sirolimus for 2 years showed an FEV_1_ decline rate of between 0.67 and 2.09 ml per month but did not differentiate between responders and non-responders^16^. One study that reported long-term effects of sirolimus in 12 patients found differences in FEV_1_ before and after treatment of − 55 ± 7 ml and − 5 ± 12 ml per year, respectively, with a follow-up time of 4.6 years on sirolimus^9^. A Chinese study which evaluated long-term sirolimus effect in 32 patients also found stabilization of pulmonary function after 4 years of sirolimus treatment^17^. It is clear that some patients treated with sirolimus do not respond to treatment and continue to lose lung function^18^. In the present series, 59% of sirolimus-treated patients were considered responders because had a mean FEV_1_ slope over 5 years of 42 ml/year vs a mean FEV_1_ slope of − 54 ml/year in the partial responders vs − 84 ml/year in the non-responders. We have also assessed the change of FEV_1_% predictive increasing in mean 2.17% in responders and dropping 2.96% in non-responders per year. In summary, of those treated for 5-years sirolimus treatment led to a clear improvement in lung function in half of the patients and had a modest effect in another 30%. Only 11% of sirolimus-treated patients had no improvement in FEV_1._

Another relevant aspect is the potential role of sirolimus as a preventive treatment of pleural complications. The present study supports the idea that sirolimus may have some preventive effect against pneumothorax and, probably, chylous effusions. Intuitively, it could be that chylothorax disappearances after sirolimus treatment might be the cause of pulmonary function improvement in those patients with chylothorax. But, chylothorax improvement is in fact secondary to sirolimus treatment. This good response of patients with chylothorax to sirolimus treatment had also been observed by Takada and colleagues^16^.

In the present series, only 9 patients treated with sirolimus were submitted to LT. The role of LT for LAM patients has evolved over time. During the 1980s, 1990s and early 2000s, the only choice for LAM patients with respiratory failure was LT, and LAM was a rare indication for LT^19^. In our series with more than 1100 LTs, LAM represents 2.5% of the total LT caseload. There was only one LAM patient who received LT and belong to CC.

The differences between the HSC and the CC, could be explained by several factors. Although the introduction of sirolimus in the treatment of LAM seems the likely main cause of these differences, other factors such as increased knowledge and interest in LAM in recent years, the development of a specialist referral centre, and earlier diagnosis with higher functional reserve could explain the better results in the CC cohort. All these factors are likely to have influenced the overall improved survival in LAM patients in the last decade and the current perception that LT in LAM will become anecdotal. Perhaps, though, this scenario is somewhat optimistic: we must add a note of caution as our data showed that sirolimus had no effect in 11% of patients with LAM at 5-years.

We observed a positive effect in most cases of angiomyolipoma after one year of treatment, but no effect in 9%. These results are in line with the study published by Bissler et al. that reported no effect in 20% of cases^10^. Also, we found that more than half of the patients who had a positive response in terms of reduction and stabilization of angiomyolipomas had a positive pulmonary functional response. Bissler and colleagues reported the same effect in 7 out of 11 patients who had PFT data at 1 year of sirolimus treatment. Davies et al. also reported, in 12 patients, a 50% decrease in angiomyolipoma size without a significant improvement in lung function during sirolimus therapy. None of the authors reported any data on a correlation between angiomyolipoma and pulmonary function response^10,20^.

Regarding safety, we recorded side effects in 33% of patients, mainly mild or moderate, and only 1 patient discontinued sirolimus due to side effects. Other authors observed the same spectrum of side effects but in 50% to 59% of patients^16,21^. We did not observe any cases of pneumonitis, something that has previously been described^21^. One of the reasons for these differences could be the retrospective design of our study in contrast with other prospective studies^16,21^. However, we consider that all serious adverse events were reported and recorded.

The main limitation of the study is its retrospective, single-centre design. The number of LAM patients included in the study was conditioned by the rarity of the disease and covers an extended period of 30 years; this is a limitation for most LAM studies. The strengths include the homogeneity of the clinical care and criteria, as most patients were treated by the same team, who had extensive experience with mTOR inhibitors, LT, and the long-term follow-up of sirolimus-treated patients.

In conclusion, sirolimus treatment in LAM has a long-term positive or moderate effect in most patients. LT remains the treatment of choice in LAM patients with respiratory failure. Determining the long-term effects of sirolimus on survival, the mechanisms that preclude treatment response and identifying potential biomarkers are important future priorities.
